# Acute myocardial infarction-related mortality among older adults (≥65 years) with malignancy in the U.S. from 1999 to 2020

**DOI:** 10.1016/j.ijcrp.2025.200392

**Published:** 2025-03-07

**Authors:** Muhammad Abdullah Naveed, Sivaram Neppala, Himaja Dutt Chigurupati, Ahila Ali, Muhammad Omer Rehan, Ayman Fath, Bazil Azeem, Rabia Iqbal, Manahil Mubeen, Hamza Naveed, Muhammad Naveed Uz Zafar, Mushood Ahmed, Jamal S. Rana, Brijesh Patel

**Affiliations:** a Department of Cardiology, Dow Medical College, Dow University of Health Sciences, Karachi, Pakistan; b Department of Cardiology, University of Texas Health Sciences Center, San Antonio, TX, USA; c Department of Internal Medicine, Saint Micheal's Medical Center, Newark, NJ, USA; d Department of Cardiology, Shaheed Mohtarma Benazir Bhutto Medical College Lyari, Karachi, Pakistan; e Department of Medicine, Queen Elizabeth the Queen Mother Hospital, EKHUFT, Margate, Kent, United Kingdom; f Department of Cardiology, Associate Professor at Liaquat University of Medical & Health Sciences, Pakistan; g Department of Medicine, Rawalpindi Medical University, Rawalpindi, Pakistan; h Department of Cardiology, The Permanente Medical Group, Oakland, CA, USA; i Indiana University School of Medicine, Indianapolis, IN, USA

**Keywords:** Acute myocardial infarction, Malignancy, Mortality, Race, Gender, Geography

## Abstract

**Background:**

Acute Myocardial Infarction (AMI) in malignancy is a global threat, causing significant mortality and economic burden. They share common risk factors, highlighting the urgency of addressing this critical issue.

**Objective:**

This study analyzed demographic trends and disparities in mortality rates due to AMI in malignancy among adults aged 65 and older from 1999 to 2020.

**Methods:**

We used the CDC WONDER database to analyze Age-adjusted mortality rates (AAMRs) for AMI in malignancy patients (ICD-10 I21, C00-C97) from 1999 to 2020, stratifying by sex, race, geography, and metropolitan status. We calculated Average Annual Percentage Changes (AAPCs) and Annual Percentage Changes (APCs) per 100,000 with 95 % confidence intervals (CI) using Joinpoint regression.

**Results:**

Between 1999 and 2020, AMI in malignancy accounted for 172,691 deaths among adults aged ≥65 years, with the majority of deaths occurring in medical facilities (56.9 %). The overall AAMR for AMI in malignancy-related deaths decreased from 30.2 in 1999 to 14.2 in 2020, with an AAPC of −3.90 (p < 0.000001). Men showed higher AAMRs than women (28.6 vs. 12.3), with a more pronounced decrease in men (AAPC: 4.22, p < 0.000001) compared to women (AAPC: 3.78, p < 0.000001). Black individuals have the highest AAMR (22.7), followed by Whites (19.3). Arkansas had the highest AAMR (32.3), while Nevada had the lowest (8.1), with the Northeastern region having the highest regional AAMR (20.2), and nonmetropolitan areas had higher AAMRs.

**Conclusion:**

This study reveals significant demographic disparities in mortality rates related to AMI in malignant older adults. These findings emphasize the need for targeted interventions and improved access to care.

## Highlights

• Our study shows a higher mortality risk from AMI in individuals aged ≥ 65 with concurrent malignancy, affecting various demographic groups.
• From 1999 to 2015, the AAMR fell significantly, with an APC of −5.23, but saw a slight increase from 2015 to 2020 of 0.45.
• Men exhibited a strikingly higher AAMR of 28.6 compared to women with an AAMR of 12.3.
• Our findings highlight significant racial disparities: white individuals have higher mortality rates, while American Indians have the lowest.
• The regional discrepancies were equally concerning, with Arizona recording the highest mortality rates and Nevada reporting the lowest AAMRs.

## 1 Introduction

Cardiovascular disease (CVD) and malignancy are prominent causes of death on a global scale, jointly contributing to nearly 70 % of disease-related fatalities in developed nations [1,2]. Both malignancy and cardiovascular diseases share risk factors such as an aging population, obesity, diabetes, and smoking [3]. Moreover, specific cancer treatments are recognized to induce cardiotoxicity, which can exacerbate cardiovascular risks through the acceleration of atherosclerosis, coronary vasospasm, or acute thrombosis [4,5]. Consequently, individuals with cancer face an increased likelihood of experiencing acute myocardial infarction (AMI).

Existing literature has outlined the detrimental impact of malignancy on the prognosis of AMI, with evidence indicating poorer outcomes in this patient demographic [6]^.^ In addition, cancer survivors may have elevated risk factors such as diabetes and hypertension compared to those without cancer [7]. Elderly individuals, precisely those aged 65 and older, are at the highest risk for both malignancy and AMI due to age-related physiological changes and the rising prevalence of chronic conditions within this group [8,9]. Nonetheless, there is a lack of comprehensive, population-level analyses that examine long-term trends in AMI-related mortality among older adults with malignancies. Understanding this mortality's demographic and geographical patterns is crucial for targeted interventions and resource allocation to enhance outcomes within this high-risk cohort.

Our study seeks to unveil a comprehensive two-decade analysis of the trends and disparities in AMI-related mortality among older U.S. adults (aged 65 and above) grappling with underlying malignancies from 1999 to 2020. Leveraging the power of the Centers for Disease Control and Prevention (CDC) WONDER database, we endeavor to illuminate the demographic and geographic factors intricately associated with AMI-related mortality in this vulnerable population. The invaluable insights and evidence derived from this study have the potential to catalyze the development of precise, efficient prevention and management strategies tailored for older adults simultaneously grappling with AMI and malignancy. Notably, this research enhances the ever-growing evidence highlighting the intricate, intertwined relationship between cardiovascular disease and oncology.

## 2 Methods

### 2.1 Study design

This research used the data obtained from the CDC WONDER database, a very reliable, comprehensive database of death certificates. Data for deaths from Acute MI in people with Malignancy were extracted for the period 1999–2020, specified by the ICD-10 codes I21.0 (Acute transmural myocardial infarction of anterior wall); I21.1 (Acute transmural myocardial infarction of inferior wall); I21.2 (Acute transmural myocardial infarction of other sites); I21.3 (Acute transmural myocardial infarction of unspecified site); I21.4 (Acute subendocardial myocardial infarction); I21.9 (Acute myocardial infarction, unspecified) for Acute MI and ICD-10 Codes C00-C97 (Malignant neoplasms) for Malignancy. Subgroup analysis utilized ICD-10 codes corresponding to specific cancer sites: Lung (C34), gastrointestinal (C15-C26), prostate (C61), breast (C50), and hematological (C81-C96). According to the latest national statistics from the United States, these cancer types were chosen to reflect the most prevalent cancers. The dataset had been used previously to research mortality trends for several diseases, which consisted of death certificates from all 50 states and the District of Columbia and concentrated on adults aged 65 years and older.

The data used in this research were de-identified, published datasets available to the public from the government. As the data were de-identified, this did not require IRB approval. However, the study follows the Strengthening the Reporting of Observational Studies in Epidemiology (STROBE) guidelines for reporting.

### 2.2 Data extraction

The data was obtained for this study on various mortality-related variables that included population size, year, place of death, demographics, urban-rural stratification, regional delineation, and specific classification to each state. All demographic data included age and race/ethnicity. The place of death was classified according to multiple classes of health care settings, such as medical facility settings like outpatient, emergency room, inpatient, death on arrival or status unknown, home, hospice, and nursing home/long-term care facility. Race/ethnicity was categorized as Hispanic and non-Hispanic White, Afro-American, Asian, or Pacific Islanders. These categorizations were made based on death certificates that had earlier been used in the CDC WONDER analysis and, thus, considered the mortal trends exhaustively and with a high level of accuracy and scrutiny.

The population was categorized according to the National Center for Health Statistics Urban-Rural Classification Scheme, which defines urban (large central metropolitan, large fringe metropolitan, medium metropolitan, and small metropolitan) and nonmetropolitan counties (micropolitan and noncore) by the 2013 US census classification for reporting the place of death. According to the US Census Bureau's definitions, regions were classified as Northeast, Midwest, South, and West.

### 2.3 Statistical analysis

The study analyzed the crude and Age-Adjusted Mortality Rate (AAMR) deaths per 100,000 individuals to investigate nationwide mortality trends. This involved determining the total number of fatalities attributed to AMI in the population with Malignancy for each year. As previously practiced, the AAMR was computed by standardizing the AMI-related deaths using the 2000 US population and 95 % confidence intervals. The annual percent change and a 95 % confidence interval for the AAMR were estimated using the JoinPoint Regression Program (Joinpoint V 4.9.0.0, National Cancer Institute, Bethesda, MD, USA). AAMRs are valid measures to compare mortality rates between different populations or periods. AAMRs were adopted in this study to underline the mortality trends and detect significant changes over time using log-linear regression models.

We thoroughly analyzed the Annual Percentage Change (APC) and Average Annual Percentage Change (AAPC) using Joinpoint regression analysis. This advanced statistical technique reveals significant shifts in trend data over time. The APC represents the annual percentage change for each segment's outcome variable, providing a detailed view of trends. Conversely, the AAPC offers a comprehensive overview of trend changes throughout the study period, effectively synthesizing the data into a cohesive narrative. The APC and AAPC are carefully reported with 95 % confidence intervals (CIs), ensuring a robust evaluation of statistical significance. Previous research utilized AAMRs, APCs, and AAPCs to uncover trends and disparities in mortality across different demographics [10,11].

## 3 Results

Between 1999 and 2020, Acute Myocardial Infarction in Malignancy accounted for a total of 172,691 deaths among older adults aged 65+ years in the United States (Supplementary Table 1). These fatalities were distributed across various settings, with the leading most occurring in medical facilities (56.9 %), 24.2 % at the decedents’ homes, 14.0 % in nursing homes/long-term care facilities, 2.1 % in hospice facilities, and 2.6 % at other locations. (Supplementary Table 2).

### 3.1 Annual trends for acute MI in malignancy-related age-adjusted mortality rate (AAMR)

The overall age-adjusted mortality rate (AAMR) for Acute MI in Malignancy-related deaths among older adults decreased from 30.2 in 1999 to 14.2 in 2020, with an Average Annual Percentage Change (AAPC) of −3.90 (95 % Confidence Interval [CI]: 4.12 to −3.68) (p-value <0.001). Notably, the AAMR experienced a significant decline from 1999 to 2015 (APC: 5.23; 95 % CI: 5.50 to −4.98) (p-value <0.001), followed by a slight increase from 2015 to 2020 (APC: 0.45; 95 % CI: 1.22 to 3.21) (p-value = 0.51). (See Supplementary Table 3).

### 3.2 Acute MI in malignancy-related AAMR Stratified by sex

Throughout the study period, older men exhibited substantially higher AAMRs than older women (overall AAMR for men: 28.6, 95 % CI: 28.4–28.8; for women: 12.3, 95 % CI: 12.2–12.4). The AAMR of both men and women decreased from 1999 to 2020, with the decrease more prominent in men [Men: AAPC: 4.22, (CI: 4.43 to −4.04) (p-value <0.001); Women: AAPC: 3.78, (CI: 4.20 to −3.48) (p-value <0.001)].

**Fig. 1:**
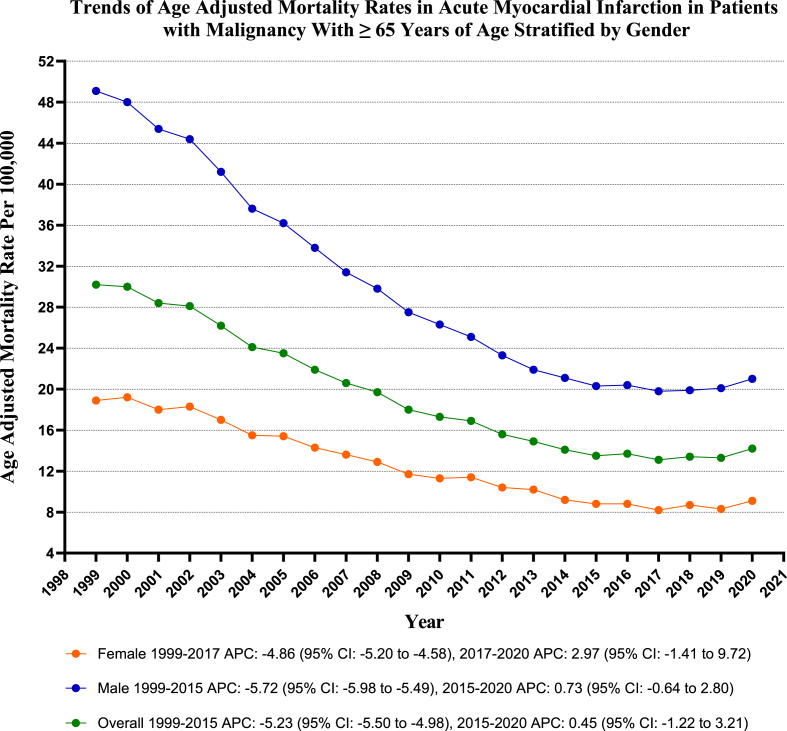
Overall and sex-Stratified deaths due to acute MI in patients with malignancy: Age-adjusted mortality rates per 100,000 in adults, United States, 1999–2020.

The AAMR for older men experienced a substantial decrease from 49.1 in 1999 to 20.3 in 2015 (APC: 5.72; 95 % CI: 5.98 to −5.49) (p-value <0.001), followed by insignificant changes between 2015 and 2020 (APC: 0.73; 95 % CI: 0.64 to 2.80) (p-value = 0.21). Similarly, the AAMR for older women exhibited a notable decrease from 18.9 in 1999 to 8.2 in 2017 (APC: 4.86; 95 % CI: 5.20 to −4.58) (p-value <0.001), followed by a considerable increase to 9.1 by 2020, which was not statistically significant (APC: 2.97; 95 % CI: 1.41 to 9.72) (p-value = 0.18). (Refer to Fig. 1 and Supplementary Table 4 for details).

### 3.3 Acute MI in malignancy-related AAMR Stratified by race/ethnicity

Significant variability in number of deaths was found among different racial/ethnic groups, with the highest number of deaths occurring in White individuals (143,709; 83.2 %), followed by Black individuals (17,266; 10.0 %), Hispanic individuals (7309; 4.2 %), Asian or Pacific Islanders (3448; 2.0 %), and the lowest number in American Indian or Alaska Native individuals (618; 0.4 %). AAMRs were highest among Black or African Americans, followed by Whites, American Indian or Alaska Natives, Hispanic or Latinos, and Asian or Pacific Islander populations (overall AAMR: Black or African American: 22.7, 95 % CI: 22.4–23.1; White: 19.3, 95 % CI: 19.2–19.4; American Indian or Alaska Native: 14.4, 95 % CI: 13.2–15.6; Hispanic or Latino: 12.2, 95 % CI: 12.0–12.5; Asian or Pacific Islander: 10.8, 95 % CI: 10.4–11.1), all p value < 0.001.

**Fig. 2:**
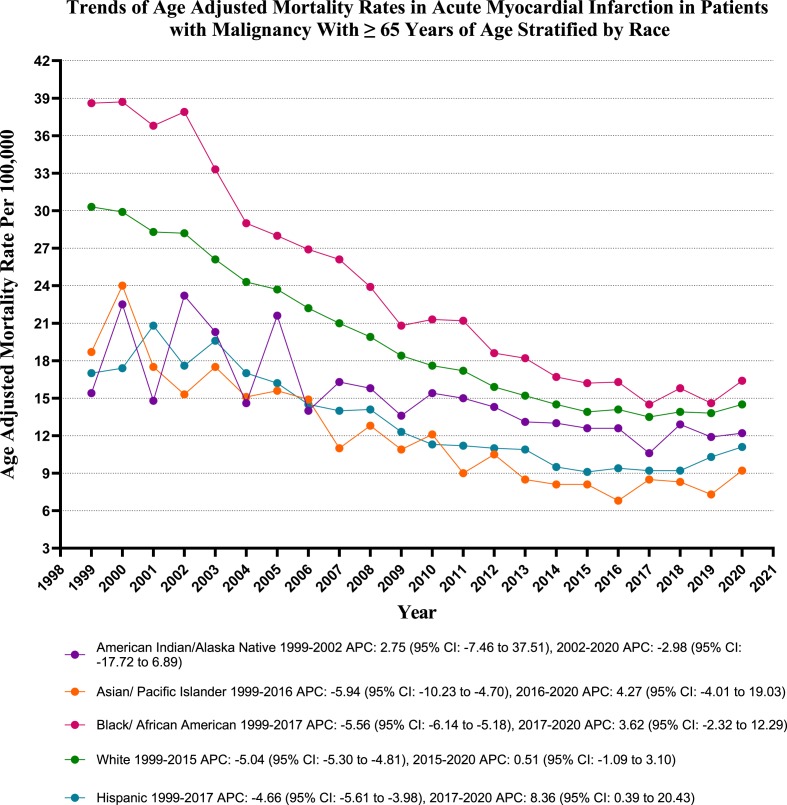
Age-adjusted mortality rates per 100,000 related to acute MI in patients with malignancy, Stratified by race in the United States, 1999 to 2020.

The AAMR of all the races decreased to variable degrees from 1999 till 2020, with the decrease most pronounced in Black [ AAPC: 4.30, 95 % CI: 4.88 to −3.84, p-value <0.001; Asian: AAPC: 4.08, 95 % CI: 5.46 to −3.14, p-value <0.001; White: AAPC: 3.57, 95 % CI: 3.87 to −3.31, p-value <0.001; Hispanic: AAPC: 2.00, 95 % CI: 2.67 to −1.13, p-value <0.001], but American individuals showed no significant changes [AAPC: 2.18, 95 % CI: 4.24 to 0.38, p-value = 0.08]. (Refer to Fig. 2 and Supplementary Table 5 for details).

### 3.4 Acute MI in malignancy-related AAMR Stratified by geographical regions

**Fig. 3:**
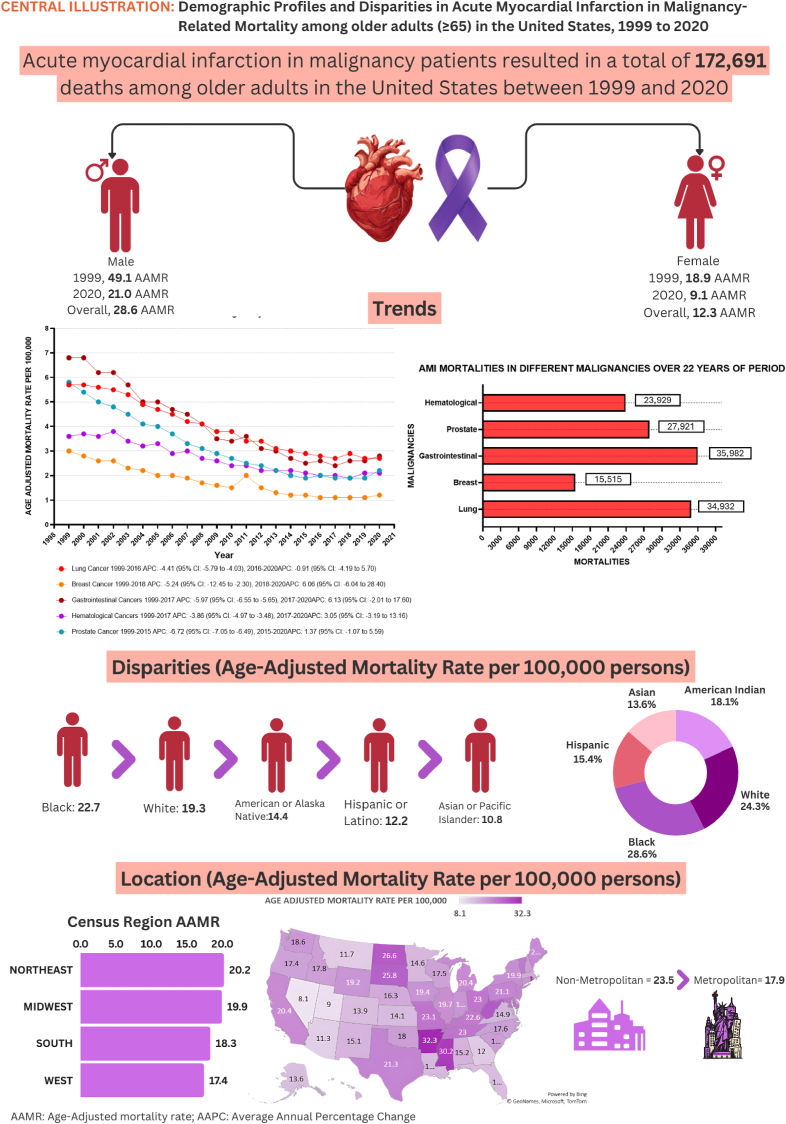
Central Illustration: Trends in demographics and disparities in acute MI among adults patients with malignancy in the United States: 1999 to 2020.

**Fig. 4:**
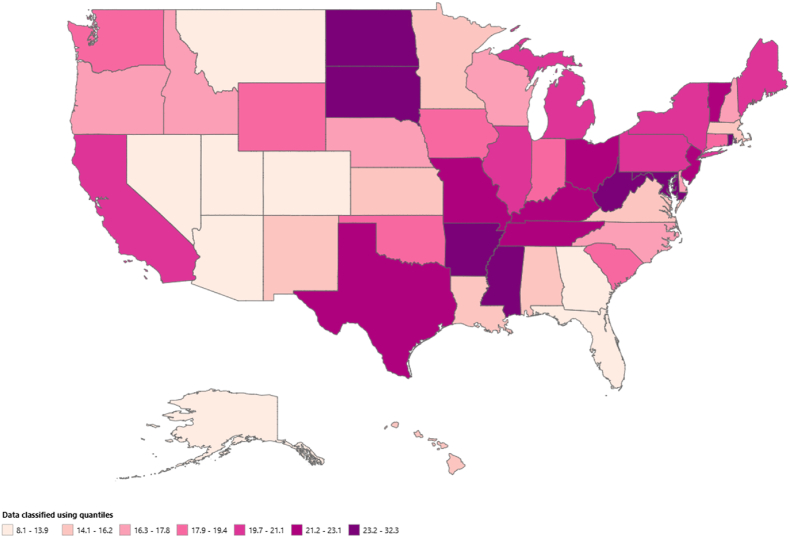
Wonder Map illustrates geographic variability trends throughout the United States, classified by quantiles.

Variations in AAMRs were observed among different states, with AAMRs ranging from as low as 8.1 (95 % CI: 7.5–8.8) in Nevada to 32.3 (95 % CI: 31.2–33.5) in Arkansas. States falling within the top 90th percentile included Arkansas, Maryland, Mississippi, Missouri, North Dakota, Rhode Island, South Dakota, and West Virginia, which had approximately one and a half times higher AAMRs compared to states in the lower 10th percentile, which included Alaska, Arizona, Colorado, Florida, Georgia, Kansas, Montana, Nevada, and Utah. (Refer to Fig. 3 and Supplementary Table 6 for details). The Wonder Map illustrates geographic variability trends throughout the United States, classified by quantiles (Fig. 4).

**Fig. 5:**
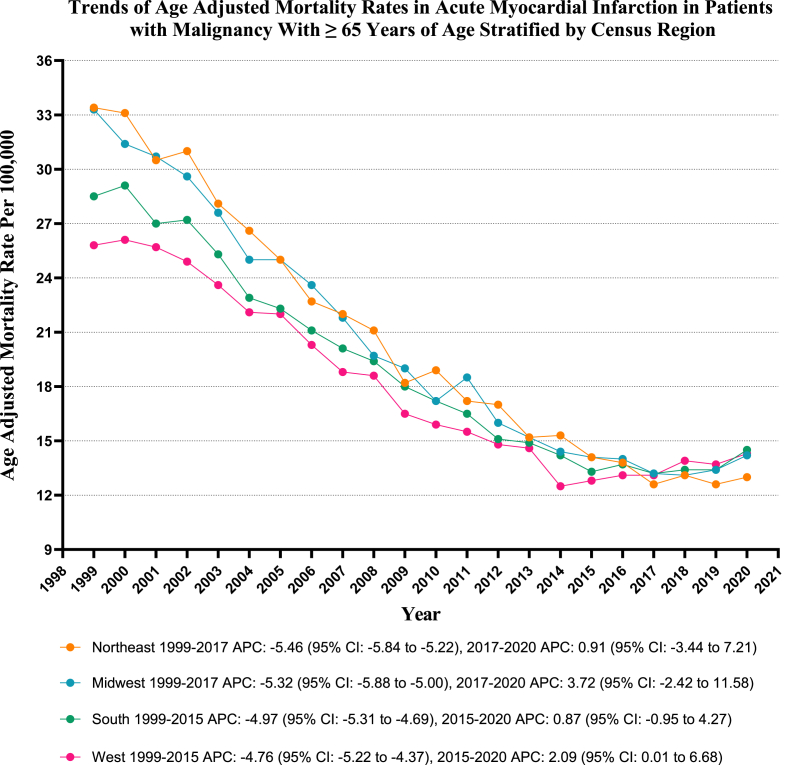
Age-adjusted mortality Rates per 100,000 Related to Acute MI in Patients with Malignancy, Stratified by Census Region in the United States, from 1999 to 2020.

On average, over the study period, the highest mortality was observed in the Northeastern (AAMR: 20.2; 95 % CI: 20.0 to 20.4), followed by the Midwestern (AAMR: 19.9; 95 % CI: 19.7 to 20.1), Southern (AAMR: 18.3; 95 % CI: 18.2 to 18.5), and Western regions (AAMR: 17.4; 95 % CI: 17.2 to 17.6), all p-value <0.001. (Refer to Fig. 5 and Supplementary Table 7 for details).

**Fig. 6:**
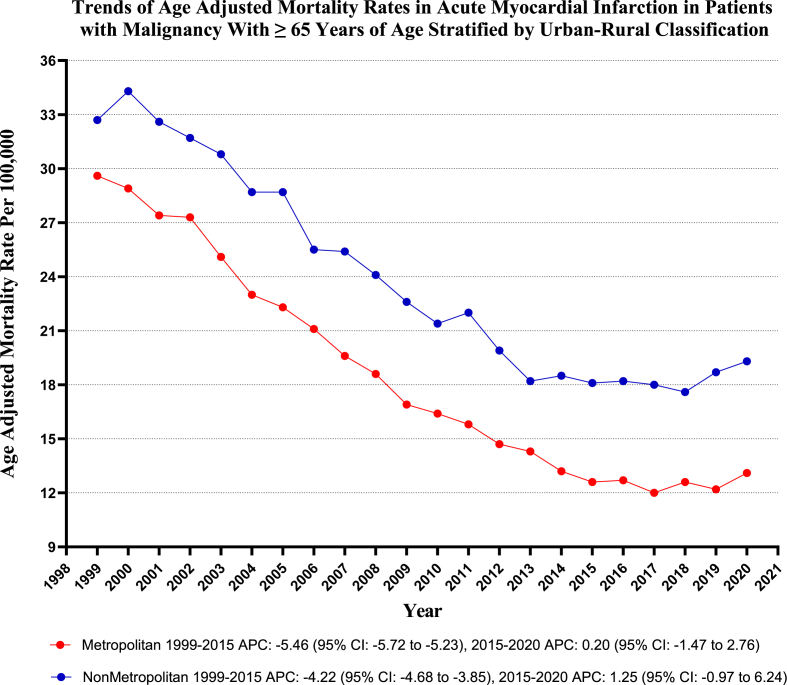
Age-adjusted mortality Rates per 100,000 Related to Acute MI in Patients with Malignancy, Stratified by Urbanization, in the United States from 1999 to 2020.

Nonmetropolitan areas exhibited slightly higher AAMRs than metropolitan areas throughout the study period, with overall AAMRs of 23.5 (95 % CI: 23.3 to 23.8) and 17.9 (95 % CI: 17.8 to 18.0), respectively. The AAMR of both Metropolitan and Nonmetropolitan areas decreased considerably from 1999 to 2020, with a more significant decrease in Metropolitan areas [Metropolitan: AAPC: 3.97, (CI: 4.21 to 2.47) (p-value <0.001); Nonmetropolitan: AAPC: 2.64, (CI: 3.04 to −2.27) (p-value <0.001)]. (Refer to Fig. 6 and Supplementary Table 8 for details).

### 3.5 Subgroup analysis

From 1999 to 2020, various malignancies exhibited a significant downward trend, albeit with some variability among them. Prostate Cancer demonstrated the steepest decline [Prostate Cancer: AAPC: 4.85, (CI: 5.23 to −4.57) (p-value <0.001); Gastrointestinal Cancers: AAPC: 4.33, (CI: 5.13 to −3.93) (p-value <0.001); Breast Cancer: AAPC: 4.22, (CI: 5.67 to −2.58) (p-value <0.001); Lung Cancer: AAPC: 3.50, (CI: 3.83 to −3.26) (p-value <0.001); Hematological Cancers: AAPC: 2.90, (CI: 3.66 to −2.29) (p-value <0.001)].

**Fig. 7:**
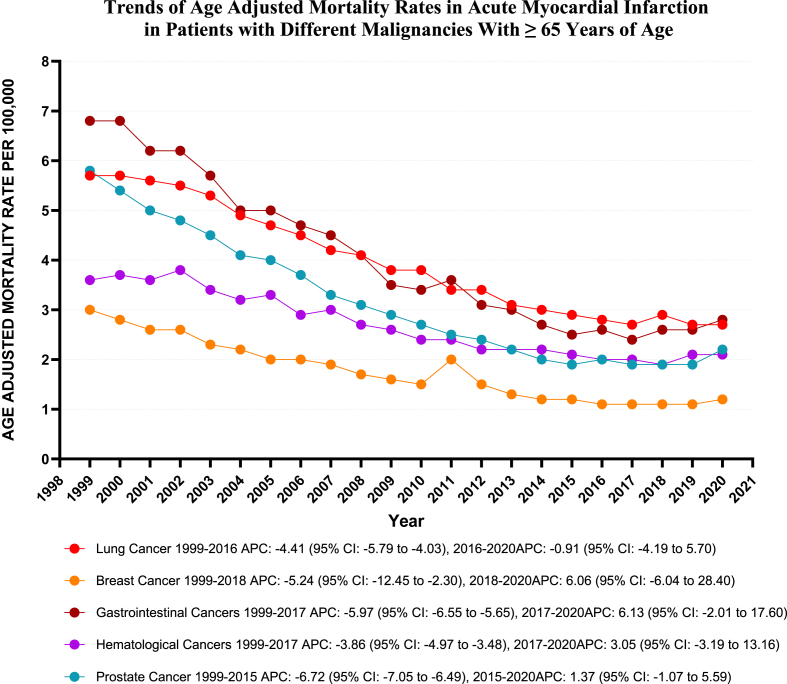
Age-adjusted mortality Rates per 100,000 Related to Acute MI in Patients with different Malignancies, Stratified by Census Region in the United States, from 1999 to 2020.

Notably, the AAMR for Gastrointestinal Cancers experienced a notable decrease from 1999 to 2017 (APC: 5.97; 95 % CI: 6.55 to −5.65) (p-value <0.001), followed by stable trends from 2017 to 2020 (APC: 6.13; 95 % CI: 2.01 to 17.60) (p-value = 0.12). Likewise, Hematological Cancers recorded a significant drop from 1999 to 2017 (APC: 3.86; 95 % CI: 4.97 to −3.48) (p-value = 0.005) but showed no significant variation from 2017 to 2020 (APC: 3.05; 95 % CI: 3.19 to 13.16) (p-value = 0.39) (Refer to Fig. 7).

## 4 Discussion

Our research provides evidence of increased mortality due to acute myocardial infarction in elderly individuals aged 65 years and older with concurrent malignancy across diverse demographic groups in the United States. Our findings revealed that between 1999 and 2020, acute myocardial infarction in malignancy resulted in 172,691 deaths among older adults aged 65 years and above in the United States. The age-adjusted mortality rate (AAMR) exhibited a significant decline from 1999 to 2015 (annual percent change (APC): 5.23), followed by a slight increase from 2015 to 2020 (APC: 0.45). Furthermore, older men had notably higher AAMRs compared to older women (AAMR: 28.6 vs. 12.3). We also observed significant racial disparities in AAMRs, with higher mortality rates among white individuals and lower rates among American Indian or Alaska Native individuals. Additionally, Arizona residents had higher AAMRs, while those in Nevada reported lower AAMRs.

Previous studies have shown that patients with cancer admitted with acute myocardial infarction face increased short-term all-cause and cardiac mortality risks, as well as elevated long-term all-cause mortality following percutaneous coronary intervention [12,13]. Furthermore, these studies have shown that elderly cancer patients exhibit higher mortality rates compared to those under 65 years of age. In our research, we observed age-adjusted mortality trends declining from 1999 to 2015 among elderly patients aged 65 years and above, but with a slight increase in mortality noted from 2015 to 2020, possibly due to the rising prevalence of obesity and other comorbidities in recent years [14]. Prior studies have highlighted a strong association between body mass index and heightened risks of various cancers, including colorectal, endometrial, ovarian, esophageal, and prostate cancers, as well as the potential impact of other overlapping risk factors such as smoking, age, hyperglycemia, alcohol use, and hyperlipidemia mediating inflammation and immune response [[15], [16], [17], [18], [19], [20], [21]]. Moreover, the progression of atherosclerotic plaques resulting from cancer therapy, particularly chemotherapy and radiation, has been linked to increased acute myocardial infarction risks [[22], [23], [24]]. Our study adds important insights for cardio-oncology and the concerted efforts in the area for improved care of patients.

Existing research has consistently pointed out the higher incidence of cardiovascular risk factors and worse health outcomes in men overall [25]. Our study reveals notable gender differences in mortality rates among patients with Acute Myocardial Infarction (AMI) and cancer, showing that older men with these conditions experience increased mortality risks compared to their female counterparts. Importantly, our findings contest earlier studies by revealing age-adjusted gender differences in mortality within this group, contradicting previous assertions of gender equality in age-adjusted mortality rates among these patients [26].

We have observed significant disparities in racial and ethnic groups among individuals with cancer and acute myocardial infarction (AMI), with the highest number of deaths occurring in White individuals, followed by Black/African American and Hispanic individuals. Previous research strongly indicates that African American individuals exhibit the highest mortality rates compared to White individuals due to the heightened prevalence of various risk factors and the influence of genetic and environmental factors [27,28]. These disparities are often linked to socioeconomic factors, inequalities in care receipt after acute coronary syndrome (ACS) among ethnic minority patients, and socioeconomic status [29,30]. Focusing on researching the racial disparities among cancer and AMI patients could provide clarity on the differences in mortality rates among these individuals.

Our research also revealed notable variations in mortality rates across different geographical regions, with elevated mortality rates predominantly observed in the northeastern and midwestern regions and with higher mortality rates in nonmetropolitan areas compared to metropolitan areas, underscoring the urgent need to address the disparity in healthcare access in rural settings. Previous research has documented marked geographical disparities, with the northeastern states experiencing declining trends while the southern regions either observed stagnant trends or higher risks [31,32]. The existing literature has also highlighted the increased mortality trends among lower socioeconomic groups and individuals residing in nonmetropolitan areas relative to their higher socioeconomic counterparts [32,33]. These evolving disparities underscore the necessity for cohesive efforts to mitigate regional discrepancies in healthcare infrastructure, socioeconomic determinants, and disease control.

## 5 Limitations

The study is subject to limitations, mainly due to its retrospective design. Relying on data from death certificates in the CDC WONDER database introduces the potential for diagnosis inaccuracies, leading to misclassification bias. Moreover, the lack of laboratory values in the medication list, baseline characteristics, and clinical data regarding general health conditions, comorbidities, and treatments inhibits a complete understanding of elevated mortality patterns. Nevertheless, we have taken steps to validate the data to ensure the credibility of our findings.

## 6 Conclusion

The study uncovered substantial demographic disparities in mortality rates associated with acute myocardial infarction (AMI) among elderly individuals with cancer. These results underscore the importance of implementing tailored interventions and enhancing healthcare access to lower mortality rates and improve outcomes for this vulnerable population.

## CRediT authorship contribution statement

**Muhammad Abdullah Naveed:** Writing – original draft, Visualization, Investigation, Formal analysis. **Sivaram Neppala:** Writing – review & editing, Writing – original draft, Supervision, Investigation, Formal analysis. **Himaja Dutt Chigurupati:** Writing – review & editing, Project administration, Conceptualization. **Ahila Ali:** Writing – original draft, Supervision, Formal analysis. **Muhammad Omer Rehan:** Validation, Formal analysis. **Ayman Fath:** Writing – review & editing, Supervision. **Bazil Azeem:** Methodology, Formal analysis. **Rabia Iqbal:** Validation, Methodology, Formal analysis. **Manahil Mubeen:** Validation, Investigation. **Hamza Naveed:** Supervision, Resources. **Muhammad Naveed Uz Zafar:** Formal analysis. **Mushood Ahmed:** Resources, Investigation, Formal analysis. **Jamal S. Rana:** Writing – review & editing, Supervision, Resources. **Brijesh Patel:** Writing – review & editing, Supervision, Resources.

## Disclosure

None

## Conflict of interest

None of the authors have any conflict of interest.
