# Supplementary material for: Acute myocardial infarction-related mortality among older adults (≥65 years) with malignancy in the U.S. from 1999 to 2020
Source: Int J Cardiol Cardiovasc Risk Prev. 2025 Mar 7;25:200392. doi: 10.1016/j.ijcrp.2025.200392 (PMC11993166; doi:10.1016/j.ijcrp.2025.200392)
Supplement: Multimedia component 1 [file mmc1.docx]

**Supplementary Tables**

**Supplemental Table 1: Acute Myocardial Infarction related mortalities in older adults with Malignancy, Stratified by Sex and Race, in the United States, 1999 to 2020**

| **Deaths** | | | | | | | | | |
| --- | --- | --- | --- | --- | --- | --- | --- | --- | --- |
| **Year** | **Overall** | **Women** | **Men** | **NH White** | **NH Black or African American** | **NH Asian or Pacific Islander** | **NH American Indian or Alaska Native** | **Hispanic or Latino** | **Population** |
| **1999** | 10,416 | 4,040 | 6,376 | 8,949 | 1,041 | 128 | 18 | 245 | 34,797,841 |
| **2000** | 10,443 | 4,137 | 6,306 | 8,899 | 1,050 | 165 | 30 | 271 | 34,991,753 |
| **2001** | 10,027 | 3,933 | 6,094 | 8,476 | 1,022 | 141 | 18 | 344 | 35,290,291 |
| **2002** | 10,029 | 3,996 | 6,033 | 8,488 | 1,054 | 133 | 30 | 305 | 35,522,207 |
| **2003** | 9,436 | 3,764 | 5,672 | 7,935 | 939 | 157 | 29 | 355 | 35,863,529 |
| **2004** | 8,777 | 3,456 | 5,321 | 7,442 | 831 | 141 | 18 | 330 | 36,203,319 |
| **2005** | 8,683 | 3,466 | 5,217 | 7,337 | 815 | 161 | 31 | 322 | 36,649,798 |
| **2006** | 8,237 | 3,243 | 4,994 | 6,940 | 796 | 164 | 21 | 307 | 37,164,107 |
| **2007** | 7,897 | 3,149 | 4,748 | 6,637 | 795 | 127 | 26 | 304 | 37,825,711 |
| **2008** | 7,672 | 3,034 | 4,638 | 6,407 | 744 | 156 | 27 | 327 | 38,777,621 |
| **2009** | 7,148 | 2,786 | 4,362 | 6,015 | 660 | 140 | 23 | 302 | 39,623,175 |
| **2010** | 6,985 | 2,737 | 4,248 | 5,810 | 692 | 159 | 27 | 286 | 40,267,984 |
| **2011** | 7,012 | 2,810 | 4,202 | 5,817 | 720 | 131 | 31 | 306 | 41,394,141 |
| **2012** | 6,679 | 2,625 | 4,054 | 5,505 | 650 | 162 | 28 | 318 | 43,145,356 |
| **2013** | 6,555 | 2,598 | 3,957 | 5,367 | 670 | 144 | 28 | 334 | 44,704,074 |
| **2014** | 6,368 | 2,436 | 3,932 | 5,228 | 642 | 145 | 30 | 313 | 46,243,211 |
| **2015** | 6,268 | 2,362 | 3,906 | 5,095 | 640 | 161 | 29 | 319 | 47,760,852 |
| **2016** | 6,483 | 2,418 | 4,065 | 5,283 | 678 | 137 | 29 | 346 | 49,244,195 |
| **2017** | 6,399 | 2,317 | 4,082 | 5,174 | 632 | 188 | 30 | 364 | 50,858,679 |
| **2018** | 6,762 | 2,518 | 4,244 | 5,434 | 708 | 190 | 39 | 376 | 52,431,193 |
| **2019** | 6,905 | 2,470 | 4,435 | 5,555 | 688 | 178 | 39 | 435 | 54,058,263 |
| **2020** | 7,510 | 2,769 | 4,741 | 5,916 | 799 | 240 | 37 | 500 | 55,659,365 |
| **Total** | **172,691** | **67,064** | **105,627** | **143,709** | **17,266** | **3,448** | **618** | **7,309** | **928,476,665** |

**Supplemental Table 2: Acute Myocardial Infarction Related Mortality, Stratified by Place of Death, in Older Adults with Malignancy in the United States, 1999 to 2020**

| **Deaths** | | | | |
| --- | --- | --- | --- | --- |
| **Year** | **Medical Facility** | **Nursing Home/Long-term Care Facility** | **Hospices** | **Home** |
| **1999** | 6,531 | 1,580 | - | 2,105 |
| **2000** | 6,585 | 1,620 | - | 2,059 |
| **2001** | 6,306 | 1,512 | - | 1,969 |
| **2002** | 6,111 | 1,600 | - | 2,067 |
| **2003** | 5,718 | 1,511 | 14 | 1,942 |
| **2004** | 5,211 | 1,334 | 25 | 1,943 |
| **2005** | 5,198 | 1,369 | 62 | 1,822 |
| **2006** | 4,831 | 1,272 | 80 | 1,849 |
| **2007** | 4,545 | 1,244 | 121 | 1,777 |
| **2008** | 4,449 | 1,076 | 144 | 1,719 |
| **2009** | 4,055 | 1,047 | 154 | 1,622 |
| **2010** | 4,031 | 945 | 163 | 1,646 |
| **2011** | 3,965 | 984 | 174 | 1,691 |
| **2012** | 3,616 | 889 | 254 | 1,698 |
| **2013** | 3,568 | 870 | 243 | 1,659 |
| **2014** | 3,410 | 820 | 267 | 1,703 |
| **2015** | 3,309 | 794 | 264 | 1,733 |
| **2016** | 3,420 | 741 | 297 | 1,859 |
| **2017** | 3,252 | 755 | 316 | 1,893 |
| **2018** | 3,358 | 778 | 340 | 2,089 |
| **2019** | 3,390 | 785 | 364 | 2,143 |
| **2020** | 3,466 | 726 | 346 | 2,734 |
| **Total** | **98,325** | **24,252** | **3,628** | **41,722** |

**Supplemental Table 3: Annual percent change (APC) of Acute Myocardial Infarction related Age-Adjusted Mortality Rates per 100,000 in Older Adults with Malignancy in the United States, 1999 to 2020**

| **Year Interval** | **APC (95% CI)** | **P-value** |
| --- | --- | --- |
| **Overall** | | |
| 1999-2002 | -3.33* (-5.16 to -0.18) | 0.044791 |
| 2002-2015 | -5.50* (-7.69 to -4.25) | 0.010798 |
| 2015-2020 | 0.85 (-0.91 to 4.11) | 0.217956 |
| **Men** | | |
| 1999-2002 | -4.05* (-5.32 to -2.05) | < 0.000001 |
| 2002-2013 | -6.17* (-7.11 to -5.94) | < 0.000001 |
| 2013-2017 | -2.44* (-5.22 to -0.97) | 0.0016 |
| 2017-2020 | 1.89* (0.25 to 4.44) | 0.028794 |
| **Women** | | |
| 1999-2017 | -4.86* (-5.20 to -4.58) | < 0.000001 |
| 2017-2020 | 2.97 (-1.41 to 9.72) | 0.186763 |
| **NH White** | | |
| 1999-2002 | -3.27* (-5.04 to -0.05) | 0.04959 |
| 2002-2015 | -5.29* (-7.75 to -2.97) | 0.017197 |
| 2015-2020 | 0.84 (-0.95 to 4.83) | 0.191962 |
| **NH Black or African American** | | |
| 1999-2017 | -5.56* (-6.14 to -5.18) | 0.0004 |
| 2017-2020 | 3.62 (-2.32 to 12.29) | 0.215557 |
| **NH American Indian or Alaska Native** | | |
| 1999-2020 | -2.68* (-3.58 to -1.64) | < 0.000001 |
| **Hispanic or Latino** | | |
| 1999-2001 | 9.54 (-1.29 to 20.50) | 0.080784 |
| 2001-2016 | -5.36* (-6.58 to -4.83) | < 0.000001 |
| 2016-2020 | 5.67* (1.59 to 16.24) | 0.012797 |
| **NH Asian or Pacific Islander** | | |
| 1999-2016 | -5.94* (-10.23 to -4.70) | 0.021196 |
| 2016-2020 | 4.27 (-4.01 to 19.03) | 0.332733 |
| **Nonmetropolitan areas** | | |
| 1999-2001 | -0.001 (-4.19 to 4.02) | 0.711458 |
| 2001-2015 | -4.48* (-7.46 to -3.37) | 0.014397 |
| 2015-2020 | 1.62 (-0.67 to 6.61) | 0.117976 |
| **Metropolitan area** | | |
| 1999-2002 | -3.71* (-5.31 to -1.27) | 0.0008 |
| 2002-2015 | -5.71* (-6.89 to -5.42) | 0.006399 |
| 2015-2020 | 0.55 (-0.92 to 2.80) | 0.358328 |
| **Northeast region** | | |
| 1999-2017 | -5.46* (-5.84 to -5.22) | < 0.000001 |
| 2017-2020 | 0.91 (-3.44 to 7.21) | 0.662268 |
| **South region** | | |
| 1999-2015 | -4.97* (-5.31 to -4.69) | < 0.000001 |
| 2015-2020 | 0.87 (-0.95 to 4.27) | 0.295941 |
| **Midwest region** | | |
| 1999-2017 | -5.32* (-5.88 to -4.98) | < 0.000001 |
| 2017-2020 | 3.72 (-2.42 to 11.58) | 0.185963 |
| **West region** | | |
| 1999-2001 | 0.48 (-3.45 to 3.37) | 0.973805 |
| 2001-2015 | -5.08* (-5.61 to -4.81) | 0.0016 |
| 2015-2020 | 2.53* (1.10 to 4.87) | 0.0004 |
| APC = annual percent change; NH = non-Hispanic; * Indicates that the annual percentage change (APC) is significantly different from zero at α = 0.05. AAMR = age-adjusted mortality rate. | | |

**Supplemental Table 4: Overall and Sex‐Stratified Acute Myocardial Infarction Related Age-Adjusted Mortality Rates per 100,000 in Older Adults with Malignancy in the United States, 1999 to 2020**

| **Age-Adjusted Rate (95% CI)** | | | |
| --- | --- | --- | --- |
| **Year** | **Men** | **Women** | **Overall** |
| **1999** | 49.1 (47.9 - 50.3) | 18.9 (18.3 - 19.5) | 30.2 (29.6 - 30.8) |
| **2000** | 48.0 (46.8 - 49.2) | 19.2 (18.6 - 19.8) | 30.0 (29.4 - 30.5) |
| **2001** | 45.4 (44.2 - 46.5) | 18.0 (17.4 - 18.6) | 28.4 (27.8 - 28.9) |
| **2002** | 44.4 (43.3 - 45.6) | 18.3 (17.7 - 18.9) | 28.1 (27.6 - 28.7) |
| **2003** | 41.2 (40.1 - 42.3) | 17.0 (16.4 - 17.5) | 26.2 (25.6 - 26.7) |
| **2004** | 37.6 (36.6 - 38.6) | 15.5 (15.0 - 16.0) | 24.1 (23.6 - 24.6) |
| **2005** | 36.2 (35.2 - 37.2) | 15.4 (14.8 - 15.9) | 23.5 (23.0 - 24.0) |
| **2006** | 33.8 (32.9 - 34.8) | 14.3 (13.8 - 14.8) | 21.9 (21.4 - 22.4) |
| **2007** | 31.4 (30.5 - 32.3) | 13.6 (13.1 - 14.1) | 20.6 (20.2 - 21.1) |
| **2008** | 29.8 (29.0 - 30.7) | 12.9 (12.5 - 13.4) | 19.7 (19.2 - 20.1) |
| **2009** | 27.5 (26.6 - 28.3) | 11.7 (11.2 - 12.1) | 18.0 (17.6 - 18.4) |
| **2010** | 26.3 (25.5 - 27.1) | 11.3 (10.9 - 11.8) | 17.3 (16.9 - 17.7) |
| **2011** | 25.1 (24.3 - 25.8) | 11.4 (10.9 - 11.8) | 16.9 (16.5 - 17.3) |
| **2012** | 23.3 (22.6 - 24.0) | 10.4 (10.0 - 10.8) | 15.6 (15.2 - 16.0) |
| **2013** | 21.9 (21.2 - 22.5) | 10.2 (9.8 - 10.6) | 14.9 (14.6 - 15.3) |
| **2014** | 21.1 (20.5 - 21.8) | 9.2 (8.8 - 9.6) | 14.1 (13.8 - 14.5) |
| **2015** | 20.3 (19.6 - 20.9) | 8.8 (8.5 - 9.2) | 13.5 (13.2 - 13.9) |
| **2016** | 20.4 (19.8 - 21.1) | 8.8 (8.5 - 9.2) | 13.7 (13.3 - 14.0) |
| **2017** | 19.8 (19.1 - 20.4) | 8.2 (7.9 - 8.5) | 13.1 (12.8 - 13.4) |
| **2018** | 19.9 (19.3 - 20.5) | 8.7 (8.4 - 9.1) | 13.4 (13.1 - 13.7) |
| **2019** | 20.1 (19.5 - 20.7) | 8.3 (8.0 - 8.6) | 13.3 (13.0 - 13.6) |
| **2020** | 21.0 (20.4 - 21.6) | 9.1 (8.8 - 9.5) | 14.2 (13.8 - 14.5) |
| **Total** | **28.6 (28.4 - 28.8)** | **12.3 (12.2 - 12.4)** | **18.9 (18.8 - 18.9)** |

**Supplemental Table 5: Race‐Stratified Acute Myocardial Infarction Related Age-Adjusted Mortality Rates per 100,000 in Older Adults with Malignancy in the United States, 1999 to 2020**

| **Age-Adjusted Rate (95% CI)** | | | | | |
| --- | --- | --- | --- | --- | --- |
| **Year** | **NH White** | **NH Black or African American** | **NH Asian or Pacific Islander** | **NH American Indian or Alaska Native** | **Hispanic or Latino** |
| **1999** | 30.3 (29.7 - 30.9) | 38.6 (36.2 - 40.9) | 18.7 (15.4 - 22.1) | 34.9 (9.3 - 25.6) | 17.0 (14.8 - 19.2) |
| **2000** | 29.9 (29.3 - 30.5) | 38.7 (36.3 - 41.0) | 24.0 (20.2 - 27.7) | 22.5 (15.0 - 32.3) | 17.4 (15.2 - 19.5) |
| **2001** | 28.3 (27.7 - 28.9) | 36.8 (34.5 - 39.0) | 17.5 (14.5 - 20.5) | 14.8 (7.9 - 21.7) | 20.8 (18.5 - 23.0) |
| **2002** | 28.2 (27.6 - 28.8) | 37.9 (35.6 - 40.2) | 15.3 (12.6 - 17.9) | 23.2 (15.5 - 33.3) | 17.6 (15.6 - 19.6) |
| **2003** | 26.1 (25.5 - 26.7) | 33.3 (31.2 - 35.4) | 17.5 (14.7 - 20.3) | 20.3 (13.5 - 29.4) | 19.6 (17.5 - 21.7) |
| **2004** | 24.3 (23.8 - 24.9) | 29.0 (27.0 - 30.9) | 15.1 (12.6 - 17.7) | 13.5 (7.2 - 19.7) | 17.0 (15.1 - 18.8) |
| **2005** | 23.7 (23.2 - 24.3) | 28.0 (26.1 - 30.0) | 15.6 (13.2 - 18.1) | 21.6 (14.4 - 31.0) | 16.2 (14.4 - 18.0) |
| **2006** | 22.2 (21.7 - 22.7) | 26.9 (25.0 - 28.7) | 14.9 (12.6 - 17.3) | 14.0 (8.5 - 21.5) | 14.5 (12.9 - 16.2) |
| **2007** | 21.0 (20.4 - 21.5) | 26.1 (24.3 - 27.9) | 11.0 (9.0 - 12.9) | 16.3 (10.5 - 24.0) | 14.0 (12.4 - 15.5) |
| **2008** | 19.9 (19.5 - 20.4) | 23.9 (22.2 - 25.6) | 12.8 (10.8 - 14.8) | 15.8 (10.3 - 23.4) | 14.1 (12.5 - 15.6) |
| **2009** | 18.4 (18.0 - 18.9) | 20.8 (19.2 - 22.4) | 10.9 (9.0 - 12.7) | 13.6 (8.5 - 20.6) | 12.3 (10.9 - 13.7) |
| **2010** | 17.6 (17.1 - 18.0) | 21.3 (19.7 - 22.9) | 12.1 (10.2 - 14.0) | 15.4 (10.0 - 22.8) | 11.3 (10.0 - 12.6) |
| **2011** | 17.2 (16.8 - 17.7) | 21.2 (19.6 - 22.7) | 9.0 (7.5 - 10.6) | 15.0 (10.1 - 21.6) | 11.2 (9.9 - 12.4) |
| **2012** | 15.9 (15.5 - 16.3) | 18.6 (17.1 - 20.0) | 10.5 (8.8 - 12.1) | 14.3 (9.4 - 20.8) | 11.0 (9.7 - 12.2) |
| **2013** | 15.2 (14.8 - 15.6) | 18.2 (16.9 - 19.6) | 8.5 (7.1 - 9.9) | 13.1 (8.6 - 19.2) | 10.9 (9.7 - 12.1) |
| **2014** | 14.5 (14.1 - 14.9) | 16.7 (15.4 - 18.0) | 8.1 (6.8 - 9.5) | 13.0 (8.6 - 18.7) | 9.5 (8.4 - 10.5) |
| **2015** | 13.9 (13.5 - 14.3) | 16.2 (15.0 - 17.5) | 8.1 (6.9 - 9.4) | 12.6 (8.3 - 18.1) | 9.1 (8.1 - 10.2) |
| **2016** | 14.1 (13.7 - 14.5) | 16.3 (15.1 - 17.6) | 6.8 (5.7 - 8.0) | 12.6 (8.4 - 18.3) | 9.4 (8.4 - 10.4) |
| **2017** | 13.5 (13.1 - 13.9) | 14.5 (13.4 - 15.7) | 8.5 (7.3 - 9.7) | 10.6 (7.0 - 15.3) | 9.2 (8.2 - 10.2) |
| **2018** | 13.9 (13.5 - 14.3) | 15.8 (14.6 - 17.0) | 8.3 (7.1 - 9.5) | 12.9 (9.0 - 17.7) | 9.2 (8.3 - 10.1) |
| **2019** | 13.8 (13.5 - 14.2) | 14.6 (13.5 - 15.8) | 7.3 (6.2 - 8.4) | 11.9 (8.4 - 16.5) | 10.3 (9.3 - 11.2) |
| **2020** | 14.5 (14.1 - 14.8) | 16.4 (15.2 - 17.6) | 9.2 (8.1 - 10.4) | 12.2 (8.5 - 16.9) | 11.1 (10.1 - 12.0) |
| **Total** | **19.3 (19.2 - 19.4)** | **22.7 (22.4 - 23.1)** | **10.8 (10.4 - 11.1)** | **14.4 (13.2 - 15.6)** | **12.2 (12.0 - 12.5)** |

**Supplemental Table 6: Acute Myocardial Infarction Related Age-Adjusted Mortality Rates per 100,000, Stratified by States, in Older Adults with Malignancy in the United States, 1999 to 2020**

| **State** | **Age-Adjusted Rate (95% CI)** |
| --- | --- |
| Alabama | 15.2 (14.6 - 15.8) |
| Alaska | 13.6 (11.4 - 15.9) |
| Arizona | 11.3 (10.8 - 11.8) |
| Arkansas | 32.3 (31.2 - 33.5) |
| California | 20.4 (20.1 - 20.6) |
| Colorado | 13.9 (13.2 - 14.5) |
| Connecticut | 19.2 (18.4 - 20.0) |
| Delaware | 17.4 (15.9 - 19.0) |
| District of Columbia | 20.8 (18.5 - 23.0) |
| Florida | 13.8 (13.5 - 14.1) |
| Georgia | 12.0 (11.6 - 12.5) |
| Hawaii | 15.3 (14.2 - 16.5) |
| Idaho | 17.8 (16.6 - 19.1) |
| Illinois | 19.7 (19.2 - 20.1) |
| Indiana | 19.3 (18.7 - 20.0) |
| Iowa | 19.4 (18.6 - 20.2) |
| Kansas | 14.1 (13.3 - 14.9) |
| Kentucky | 22.6 (21.8 - 23.4) |
| Louisiana | 14.3 (13.6 - 15.0) |
| Maine | 20.5 (19.2 - 21.7) |
| Maryland | 23.2 (22.4 - 23.9) |
| Massachusetts | 16.2 (15.7 - 16.8) |
| Michigan | 20.4 (19.8 - 20.9) |
| Minnesota | 14.6 (14.0 - 15.2) |
| Mississippi | 30.2 (29.0 - 31.4) |
| Missouri | 23.1 (22.4 - 23.8) |
| Montana | 11.7 (10.5 - 12.8) |
| Nebraska | 16.3 (15.3 - 17.4) |
| Nevada | 8.1 (7.5 - 8.8) |
| New Hampshire | 17.8 (16.5 - 19.1) |
| New Jersey | 21.2 (20.6 - 21.7) |
| New Mexico | 15.1 (14.1 - 16.1) |
| New York | 19.9 (19.6 - 20.3) |
| North Carolina | 17.6 (17.1 - 18.1) |
| North Dakota | 26.6 (24.5 - 28.7) |
| Ohio | 23.0 (22.5 - 23.5) |
| Oklahoma | 18.0 (17.2 - 18.8) |
| Oregon | 17.4 (16.6 - 18.1) |
| Pennsylvania | 21.1 (20.6 - 21.5) |
| Rhode Island | 31.3 (29.5 - 33.2) |
| South Carolina | 17.9 (17.2 - 18.6) |
| South Dakota | 25.8 (23.9 - 27.7) |
| Tennessee | 23.0 (22.4 - 23.7) |
| Texas | 21.3 (20.9 - 21.6) |
| Utah | 9.0 (8.2 - 9.8) |
| Vermont | 22.7 (20.6 - 24.8) |
| Virginia | 14.9 (14.4 - 15.4) |
| Washington | 18.6 (18.0 - 19.2) |
| West Virginia | 26.7 (25.5 - 28.0) |
| Wisconsin | 17.5 (16.9 - 18.1) |
| Wyoming | 19.2 (17.0 - 21.4) |

**Supplemental Table 7: Acute Myocardial Infarction Related Age-Adjusted Mortality Rates per 100,000, Stratified by Census Region, in Older Adults with Malignancy in the United States, 1999 to 2020**

|  | **Census Region: Northeast** | **Census Region: Midwest** | **Census Region: South** | **Census Region: West** |
| --- | --- | --- | --- | --- |
| **Year** | **Age-Adjusted Rate (95% CI)** | **Age-Adjusted Rate (95% CI)** | **Age-Adjusted Rate (95% CI)** | **Age-Adjusted Rate (95% CI)** |
| **1999** | 33.4 (32.1 - 34.7) | 33.3 (32.0 - 34.5) | 28.5 (27.5 - 29.4) | 25.8 (24.6 - 27.0) |
| **2000** | 33.1 (31.8 - 34.4) | 31.4 (30.2 - 32.6) | 29.1 (28.2 - 30.1) | 26.1 (24.9 - 27.3) |
| **2001** | 30.5 (29.3 - 31.8) | 30.7 (29.5 - 31.9) | 27.0 (26.1 - 28.0) | 25.7 (24.5 - 26.9) |
| **2002** | 31.0 (29.8 - 32.3) | 29.6 (28.4 - 30.7) | 27.2 (26.3 - 28.1) | 24.9 (23.8 - 26.1) |
| **2003** | 28.1 (26.9 - 29.3) | 27.6 (26.5 - 28.7) | 25.3 (24.4 - 26.2) | 23.6 (22.5 - 24.8) |
| **2004** | 26.6 (25.5 - 27.8) | 25.0 (24.0 - 26.1) | 22.9 (22.1 - 23.8) | 22.1 (21.0 - 23.1) |
| **2005** | 25.0 (23.9 - 26.1) | 25.0 (23.9 - 26.0) | 22.3 (21.5 - 23.1) | 22.0 (21.0 - 23.1) |
| **2006** | 22.7 (21.7 - 23.8) | 23.6 (22.6 - 24.7) | 21.1 (20.4 - 21.9) | 20.3 (19.3 - 21.3) |
| **2007** | 22.0 (20.9 - 23.0) | 21.8 (20.9 - 22.8) | 20.1 (19.3 - 20.8) | 18.8 (17.9 - 19.8) |
| **2008** | 21.1 (20.1 - 22.2) | 19.7 (18.8 - 20.7) | 19.4 (18.7 - 20.1) | 18.6 (17.7 - 19.5) |
| **2009** | 18.2 (17.3 - 19.2) | 19.0 (18.1 - 19.9) | 18.0 (17.3 - 18.7) | 16.5 (15.7 - 17.4) |
| **2010** | 18.9 (18.0 - 19.9) | 17.2 (16.4 - 18.1) | 17.2 (16.6 - 17.9) | 15.9 (15.0 - 16.7) |
| **2011** | 17.2 (16.3 - 18.1) | 18.5 (17.6 - 19.3) | 16.5 (15.9 - 17.2) | 15.5 (14.7 - 16.3) |
| **2012** | 17.0 (16.1 - 17.8) | 16.0 (15.2 - 16.8) | 15.1 (14.5 - 15.7) | 14.8 (14.0 - 15.6) |
| **2013** | 15.2 (14.3 - 16.0) | 15.2 (14.4 - 15.9) | 14.9 (14.3 - 15.5) | 14.6 (13.8 - 15.4) |
| **2014** | 15.3 (14.4 - 16.1) | 14.4 (13.7 - 15.2) | 14.2 (13.6 - 14.8) | 12.5 (11.8 - 13.2) |
| **2015** | 14.1 (13.3 - 14.9) | 14.1 (13.3 - 14.8) | 13.3 (12.7 - 13.8) | 12.8 (12.1 - 13.5) |
| **2016** | 13.8 (13.0 - 14.6) | 14.0 (13.3 - 14.7) | 13.7 (13.1 - 14.2) | 13.1 (12.4 - 13.8) |
| **2017** | 12.6 (11.8 - 13.3) | 13.2 (12.5 - 13.8) | 13.2 (12.6 - 13.7) | 13.1 (12.4 - 13.8) |
| **2018** | 13.1 (12.4 - 13.9) | 13.1 (12.4 - 13.8) | 13.4 (12.9 - 14.0) | 13.9 (13.2 - 14.6) |
| **2019** | 12.6 (11.9 - 13.4) | 13.4 (12.7 - 14.1) | 13.4 (12.9 - 13.9) | 13.7 (13.0 - 14.3) |
| **2020** | 13.0 (12.3 - 13.7) | 14.2 (13.5 - 14.9) | 14.5 (14.0 - 15.0) | 14.3 (13.6 - 15.0) |
| **Total** | **20.2 (20.0 - 20.4)** | **19.9 (19.7 - 20.1)** | **18.3 (18.2 - 18.5)** | **17.4 (17.2 - 17.6)** |

**Supplemental Table 8: Acute Myocardial Infarction Related Age-Adjusted Mortality Rates per 100,000, Stratified by Urban-Rural Classification, in Older Adults with Malignancy in the United States, 1999 to 2020**

| **Age-Adjusted Rate (95% CI)** | | |
| --- | --- | --- |
| **Year** | **Metropolitan** | **Nonmetropolitan** |
| **1999** | 29.6 (29.0 - 30.2) | 32.7 (31.3 - 34.0) |
| **2000** | 28.9 (28.3 - 29.6) | 34.3 (32.9 - 35.7) |
| **2001** | 27.4 (26.8 - 28.0) | 32.6 (31.2 - 33.9) |
| **2002** | 27.3 (26.7 - 27.9) | 31.7 (30.3 - 33.0) |
| **2003** | 25.1 (24.5 - 25.6) | 30.8 (29.5 - 32.2) |
| **2004** | 23.0 (22.5 - 23.6) | 28.7 (27.4 - 29.9) |
| **2005** | 22.3 (21.7 - 22.8) | 28.7 (27.4 - 30.0) |
| **2006** | 21.1 (20.5 - 21.6) | 25.5 (24.3 - 26.6) |
| **2007** | 19.6 (19.1 - 20.1) | 25.4 (24.2 - 26.6) |
| **2008** | 18.6 (18.2 - 19.1) | 24.1 (22.9 - 25.2) |
| **2009** | 16.9 (16.5 - 17.4) | 22.6 (21.5 - 23.7) |
| **2010** | 16.4 (16.0 - 16.8) | 21.4 (20.4 - 22.5) |
| **2011** | 15.8 (15.3 - 16.2) | 22.0 (20.9 - 23.0) |
| **2012** | 14.7 (14.3 - 15.1) | 19.9 (18.9 - 20.9) |
| **2013** | 14.3 (13.9 - 14.7) | 18.2 (17.2 - 19.1) |
| **2014** | 13.2 (12.8 - 13.5) | 18.5 (17.6 - 19.5) |
| **2015** | 12.6 (12.2 - 12.9) | 18.1 (17.2 - 19.1) |
| **2016** | 12.7 (12.3 - 13.1) | 18.2 (17.3 - 19.2) |
| **2017** | 12.0 (11.7 - 12.4) | 18.0 (17.1 - 19.0) |
| **2018** | 12.6 (12.2 - 12.9) | 17.6 (16.7 - 18.5) |
| **2019** | 12.2 (11.9 - 12.6) | 18.7 (17.8 - 19.6) |
| **2020** | 13.1 (12.7 - 13.4) | 19.3 (18.4 - 20.2) |
| **Total** | **17.9 (17.8 - 18.0)** | **23.5 (23.3 - 23.8)** |
